# Effectiveness of XP-Endo Finisher and Passive Ultrasonic Irrigation in the Removal of the Smear Layer Using two Different Chelating Agents

**DOI:** 10.30476/DENTJODS.2021.86680.1204

**Published:** 2021-12

**Authors:** Ismael Espinoza, Antonio Jesus Conde Villar, Gaizka Loroño, Roberto Estevez, Gianluca Plotino, Rafael Cisneros

**Affiliations:** 1 Postgraduate Program in Endodontics, European University of Madrid, Madrid, Spain; 2 Private Practice, Grande Plotino & Torsello-Studio di Odontoiatria, Rome, Italy

**Keywords:** Smear layer, Passive ultrasonic irrigation, Xp-endo Finisher, Scanning electron microscopy

## Abstract

**Statement of the Problem:**

The smear layer may harbor microorganisms and necrotic pulp tissue, jeopardizing irrigant penetration. Recently, Dual Rinse^®^, a weak chelating agent, has been introduced to the market. However, its chelating capacity in the final irrigation protocol with different activation systems has not yet been deeply analyzed.

**Purpose:**

The aim of this *ex vivo* study was to evaluate the effectiveness of passive ultrasonic irrigation (PUI) and XP-endo Finisher (XP) on smear layer removal in combination with two chelating agents, ethylenediaminetetraacetic acid (EDTA) and etidronic acid (HEDP).

**Materials and Method:**

This *in vitro*, experimental study evaluated fifty-two single-rooted human teeth were standardized to 16 mm in length. Root canal instrumentation was performed by the ProTaper Gold system up to the F4 file. The apical end of the samples was sealed with wax to simulate a closed system. Teeth from group 1 (n=24) were irrigated with 3% sodium hypochlorite (NaOCl) and 17% EDTA, while teeth from group 2 (n=24) were irrigated with 3% NaOCl mixed 9% HEDP. Both groups were divided into two subgroups (n=12) depending on the activation system used: XP (group XP-EDTA and XP-HEDP) or PUI (group PUI-EDTA and PUI-HEDP). The specimens were evaluated by scanning electron microscopy at 3, 5 and 8mm from the apex. Statistical analysis was performed using ANOVA and Bonferroni tests considering *p*> 0.05 as significant.

**Results:**

PUI-EDTA was the most effective at removing the smear layer, with a statistically significant difference from XP-EDTA (*p*< 0.042) and group XP-HEDP (*p*< 0.003). There were no statistically significant differences among the other groups.

**Conclusion:**

Under the conditions of this *ex vivo* study, no activation system was able to completely remove the smear layer from the root canal walls. However, the combination of NaOCl with ultrasonically activated EDTA obtained better results than the other treatments.

## Introduction

An irregular layer containing microorganisms, organic and inorganic remnants and odontoblastic processes is formed by action of instruments that covers the instrumented walls [ 1 ]. This smear layer reduces the ability of the irrigants to penetrate the dentinal tubules [ 2 ] and may prevent the adaptation of filling materials to the dentinal walls [ 3 ]. In addition, it may serve a nutrient for residual bacteria inside the root canal system after endodontic procedures, thus promoting treatment failure [ 4 ].

Although sodium hypochlorite (NaOCl) has most of the properties required for an irrigant, it is not capable of removing the inorganic part of the smear layer [ 5 ]. The chelators, such as ethylenediaminetetraacetic acid (EDTA) and citric acid, may play an important role in smear layer removal during endodontic treatment [ 6 ]. Although both solutions are capable of removing the smear layer, citric acid has a higher capacity at equal concentrations [ 7 ]. Chelating agents react with the calcium ions of the debris that are formed during the instrumentation of the canal, keeping the particles in suspension and facilitating their removal. 

During instrumentation, it is recommended to avoid mixing NaOCl with the chelating agent because their combination can weaken dentin and affect its integrity [ 8 ]. Furthermore, the mixture of NaOCl+EDTA decreases the amount of available chlorine and consequently the antimicrobial capacity of the NaOCl solution [ 9 - 10 ]. Recently, a weaker chelator known by the name etidronic acid (HEDP) has been introduced to the market (Dual Rinse, Medcem, Weinfelden, Switzerland). HEDP mixed with NaOCl is capable of maintaining the properties of chlorine, does not affect the dissolving capacity, does not decrease the antimicrobial activity and simultaneously removes the smear layer [ 11 - 13 ].

Positive pressure syringe irrigation is the most commonly used irrigation system [ 14 ]. Due to its limitations [ 15 - 17 ] and difficulties in addressing complex morphologies [ 18 ] it is recommended to combine syringe irrigation with an activation system to increase the effectiveness of the solutions [ 19 - 20 ]. Furthermore, the ability enhancing efficacy of passive ultrasonic irrigation (PUI) to enhance the efficacy of chelating agents has been demonstrated [ 21 - 23 ].

Recently, a non-tapered nickel-titanium rotary instrument (XP-endo Finisher; FKG, La Chaux-de-Fonds, Switzerland) has been designed specifically to increase the efficiency in root canal wall cleaning [ 24 ] with limited impact on dentine. The XP-endo Finisher (XP) is made of a proprietary heat-treated NiTi MaxWire alloy (Martensite-Austenite Electropolish-FleX) with a transition temperature near body temperature which permits the file to change its shape to the austenite phase, allowing the file to expand its reach to 6 mm in diameter [ 25 ]. Throughout the literature, the efficacy of XP in reducing microorganisms [ 26 - 27 ], dissolving organic tissue from artificial cavities in combination with NaOCl [ 28 ] and penetrating into the isthmus [ 29 ] has been analyzed. In addition, the capacity of XP in conjunction with different irrigants to the remove the smear layer and debris has been studied [ 30 - 34 ]. However, its combination with a weak chelator has not been analyzed deeply, particularly adjunct with HEDP and a standardized and constant flow rate monitoring. For that reason, this *ex vivo* study aimed to evaluate smear layer removal using a standardized flow rate with two different chelating agents (EDTA and HEDP) and two different activation techniques (XP and PUI).

## Materials and Method

This study was carried out taking into consideration the World Medical Association *Declaration of Helsinki* (version VI, 2002) and the requirements of Spanish legislation. 

Based on information published in a previous article Lee *et al*. [ 35 ], a power calculation was carried out through the chi-squared test family and variance statistical test (G*Power 3.1 software; Heinrich Heine University, Dusseldorf, Germany) with a= 0.05 and b= 0.95. The minimum sample size was established at n=12.

The instrumentation was carried out by an experienced endodontist and methodology was carried out according to 2 previously described protocols [ 36 - 37 ]. Fifty-two recently extracted maxillary central incisor teeth of similar length and dimensions were inspected through a dental operating microscope (OPMI Pico Dental Microscope, Carl Zeiss, Oberkochen, Germany) with 20× magnification with the aim of excluding those with open apices, resorptive defects and longitudinal fractures. Teeth included in this study were stored at 6°C in a water solution containing 0.2% thymol, cleaned ultrasonically and used within a maximum of 30 days after their extraction. For specimen standardization, the crowns were removed with a high-speed long-tapered chamfer diamond bur (Komet Dental, Lemgo, North Rhine-Westphalia, Germany) obtaining lengths of 16 mm verified using a 150mm digital caliper (Vernier, Software & Technology, Beaverton, OR, USA). A stainless-steel size 10 K-File (Dentsply- Maillefer, Ballaigues, Switzerland) was introduced in the root canals until it was visible al the level of the apical foramen under magnification. The working length was calculated by reducing this length by 1 mm. 

Consequently, the apical two-thirds of the specimens were sealed with wax to simulate a closed system and included in a hydrophilic vinyl-polysiloxane regular body impression material matrix (Garant Imprint II, 3M ESPE, Madrid, Spain) to make a customized model for each sample. Following the preparation of a manual glide path working with size 15 and 20 K-Files, the root canals were instrumented up to an F4 instrument of ProTaper Gold system (Dentsply-Maillefer) following the manufacturer's recommendations. After the preparation of a manual glide path working with size 15 and 20 K-Files, teeth were divided into 4 groups (n=12) depending on the chelating agent and activation system: XP-EDTA, XP-HEDP, PUI-EDTA and PUI-HEDP.

**XP-EDTA group (n=12):** Root canal shaping was carried out up to an F4 instrument of ProTaper Gold system (Dentsply-Maillefer) agreement to the manufacturer’s recommendations. During instrumentation, after each file usage, 1.5 mL of 3% NaOCl (CanalPro, Coltene Whaledent, Altstätten, Switzerland) was used during 30 seconds with a syringe and a side-vented 27 gauge endodontic needle (Monoject, Tyco Healthcare, Mettawa, IL, USA) positioned 2 mm short of its binding point and never deeper than 2 mm from the working length. A programmable syringe pump (NE-300 ‘Just infusion’, New Era Pump Systems Inc, Farmingdale, NY, USA) at 3 mL/min was used for all irrigation procedures to avoid flow rate pauses and drop offs during irrigation procedure. Then, the following final irrigation regimen was carried out: irrigation with 1mL of 3% NaOCl for 30 seconds and activation with XP for 30 seconds; irrigation with 1mL of 17% EDTA (Dentaflux, Madrid, Spain) for 30 seconds and activation of the solution for 30 seconds; and a final flush using 1mL of 3% NaOCl for 30 seconds. XP activation was performed at 800 rpm and 1 N/cm of torque, with gentle and smooth up and down movements of 7-8 mm after reaching the working length as indicated by the manufacturer. The total irrigant volume used was 12 mL.

**XP-HEDP group (n=12):** The chemomechanical preparation was identical to that in the XP-EDTA group but a mixture of 10 mL of 3% NaOCl with 0.9 g of Dual Rinse (Dual Rinse^®^ HEDP, Medcem, Weinfelden, Switzerland) was used following the manufacturer´s recommendations was used. The final irrigation regimen was as follows: irrigation with 1mL of NaOCl + HEDP mixture for 30 seconds and activation of the solution for 30 seconds with XP repeated twice, and a final flush using 1 mL of NaOCl + HEDP for 30 seconds.

PUI-EDTA group (n=12): The chemomechanical preparation and the final irrigation regimen were the same as those in the XP-EDTA group, with the exception of the use of PUI instead of XP. PUI was performed using an ultrasonic file with a tip size of 20, no taper and 21mm length (Irri-Safe, Satelec-Acteon, Merignac, France) mounted on an ultrasonic device (P5 Newton unit, Satelec Acteon) at a power setting of 4 and placed 2mm from the working length.

**PUI-HEDP group (n=12):** The chemomechanical preparation and the final irrigation regimen were the same as in the XP-HEDP group, but PUI was used as previously described instead of XP.

Twelve teeth were used as a control group in which the same amount of distilled water was used for irrigation during and after the instrumentation procedures.

All samples were then stored at 6°C in water solution containing 0.2% of thymol, and longitudinal grooves were created on the roots using dental microscope with a double-sided diamond disc in 0.1 mm thickness (Komet Dental, Lemgo North Rhine-Westphalia, Germany) mounted on a laboratory handpiece without reaching the root canal. Subsequently, the roots were separated into two parts using a disposable scalpel blade nº 15 (Hu-Friedy, Tuttlingen, Germany). Each half sample was then analyzed by a scanning electron microscope (SEM; Jeol JSM-5200 Tokyo, Japan) in the apical, middle and coronal thirds of the canal (at 2, 5 and 8 mm respectively) at 100X and 800X magnification. The area corresponding with the greatest amount of smear layer was photographed [ 37 ]. Each root third was evaluated for smear layer removal by two experienced examiners based on the following scale [ 35 ]: 0, all the tubules were visible; 1, more than 50% of the tubules were visible; 2, less than 50% of the tubules were visible; s 3, no tubules were visible.

The mean and standard deviation were then calculated for each group and statistical analysis of the data was carried out by SPSS 21.0 (SPSS Inc, Chicago, IL) program. ANOVA and Bonferroni tests were used, with statistical significance established at *p*<.05. 

## Results

**Figure 1:**
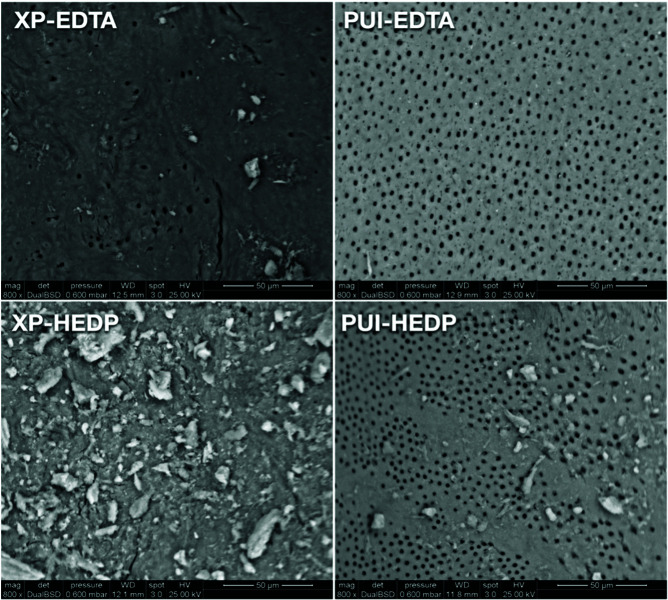
Representative scanning electron microscopy images of the apical third for the XP-EDTA, PUI-EDTA, XP-HEDP and PUI-HEDP groups

The interexaminer agreement was 87.3%, as demonstrated the kappa test. The mean scores and standard deviation of all tested groups are reported in Table 1. Scanning electron microscopy images taken after the different irrigation protocols in the different root canal thirds are shown in Figures 1 [Fig JDS-22-243-g002.tif]-3. In the control specimens, the smear layer was not removed from the root canal walls, and the scores resulted significantly different compared with those of all tested groups at all canal levels (*p*<.05). None of the tested groups was able to fully eliminate the smear layer from the canal walls except in the coronal third of groups PUI-EDTA and PUI-HEDP. 

**Table 1:** Mean± standard deviation of the scores indicating the smear layer removal in the different experimental groups and the different root canal thirds. Different superscript letters indicate a statistically significant difference

	Apical third	Middle third	Coronal third	Total
XP-EDTA	1.5±1.16^ab^	1.4±0.79^a^	0.83±0.83^b^	1.2±0.92^b^
PUI-EDTA	0.8±0.78^a^	0.4±0.69^a^	0±0^a^	0.4±0.49^a^
XP-HEDP	2.25±1.05^b^	1.4±1.08^a^	0.9±1.08^b^	1.5±1.07^b^
PUI-HEDP	1.25±1.13^ab^	0.8±0.96^a^	0±0^a^	0.68±0.69^ab^
Control Group	4±0^c^	4±0^c^	4±0^c^	12±0^c^

**Figure 2 JDS-22-243-g002.tif:**
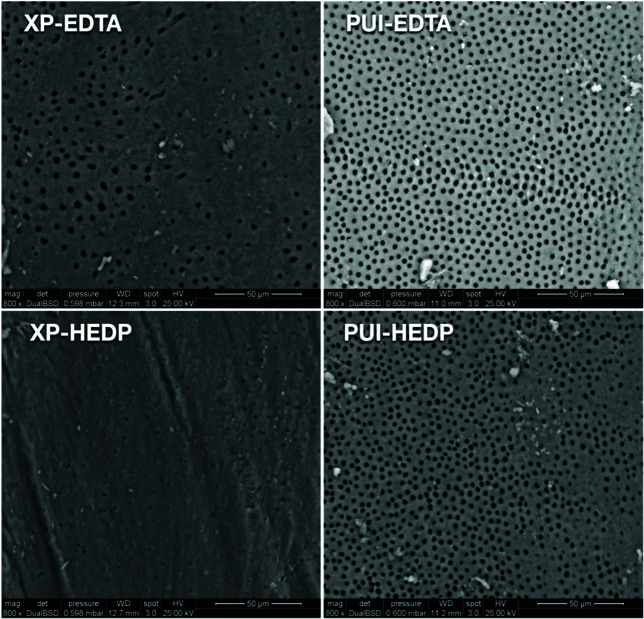
Representative scanning electron microscopic images of the middle third for the XP-EDTA (c), PUI-EDTA (a), XP-HEDP (d) and PUI-HEDP (b) groups

**Figure 3:**
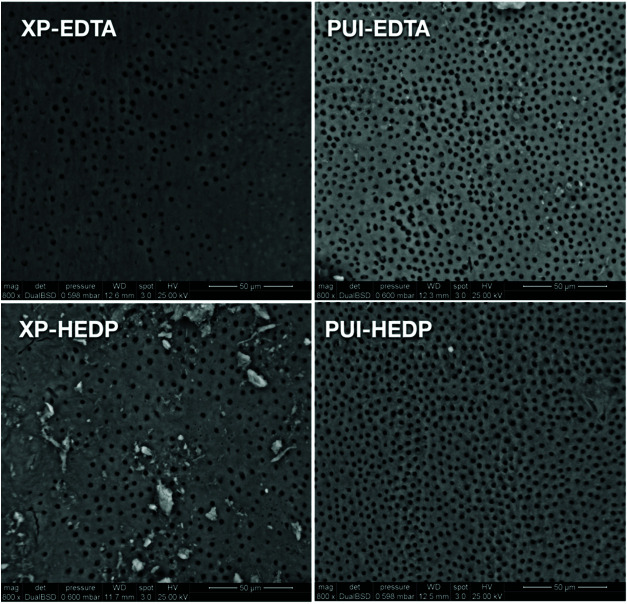
Representative scanning electron microscopic images of the coronal third for the XP-EDTA, PUI-EDTA, XP-HEDP and PUI-HEDP groups

The statistical analysis showed that group PUI-EDTA removed significantly more smear layers than group XP-EDTA and group XP-HEDP (*p*<.05). There was no statistically significant difference between the other groups (*p*> .05). The amount of the smear layer removed in the apical third was significantly greater in the PUI-EDTA group than in the XP-HEDP group (*p*< .05). However, there was no statistically significant difference between the other groups (*p*> .05). In the middle third, there was no statistically significant difference between any pair of groups (*p*> .05), while in the coronal third group PUI-EDTA and PUI-HEDP removed more of the smear layer than did XP-EDTA and XP-HEDP (*p*< .05).

## Discussion

The aim of this research was to analyze the effectiveness of two different chelating agents activated with two different techniques in the elimination of the smear layer. Although it is difficult to standardize irrigation with two chelating agents that are applied at different treatment times, in the present study, the same amount of irrigant (12mL) was used during both chemomechanical preparation and the final irrigation protocol and for the same interval of time. Moreover, to our knowledge, this is the only study involving HEDP that uses standardized constant flow rate irrigation with a positive pressure needle. A clinically realistic flow rate of 3mL/ min was used [ 38 ].

The most frequently used endodontic solutions are NaOCl, EDTA and chlorhexidine, although neither of them alone exhibits all ideal properties [ 5 ]. There are controversies related to the concentration of NaOCl that should be used, how irrigants affect to the properties of dentine and what clinicians should do when mixing the solutions [ 39 ]. Regarding chelators used in endodontics, in a poll carried out in the USA, 80% of the individuals surveyed used EDTA, 16% MTAD and lower percentages other agents such as citric acid [ 40 ]. HEDP has been introduced recently as a weaker chelating agent that can be used during root canal instrumentation due to its compatibility with NaOCl [ 12 ] and has demonstrated promising results [ 33 ]. However, its efficacy in the smear layer removal during chemomechanical preparation and in conjunction with different activation devices has not been analyzed. It must be taken into consideration that the effects of HEDP are dependent on concentration, so higher concentrations could produce different results [ 11 ].

The findings of the present study revealed that the null hypothesis may be rejected. They confirmed those published previously demonstrating that HEDP had a lower chelating capacity than EDTA [ 7 , 11 ]. In fact, under the conditions of this study, the use of HEDP during chemomechanical preparation does not prevent smear layer formation. 

However, the debate about the ideal chelator remains open, taking into account that the strong chelators, such as citric acid and EDTA, present certain disadvantages. Strong chelators may compromise the mechanical integrity of dentine and erode the dentinal tubules [ 41 - 42 ] and may negatively affect the antimicrobial and solvent properties of NaOCl if they are mixed [ 12 ]. Moreover, they are not able to completely eliminate the accumulated debris [ 43 ], and an increase in preparation errors has been reported when they were used during instrumentation [ 44 ]. Therefore, the use of HEDP during the instrumentation step [ 33 ] and a final activation using a strong chelating agent may represent an interesting combination to prevent the storage of debris and eliminate the smear layer more effectively. Future studies are necessary to investigate the efficacy of this mixing on the reduction of accumulated debris and elimination of the smear layer. However, to be able to use only one irrigant during the entire treatment process, future studies should also evaluate whether a longer final activation time of HEDP may be able to achieve the same efficacy as EDTA.

Recently, XP has been introduced as a supplementary technique to be used as a final step to improve the efficacy of root canal cleaning and disinfection [ 45 ]. Although a final activation using PUI may improve root canal cleaning [ 36 ], this technique is not able to completely clean and disinfect the entire root canal system [ 43 ]. In this study, the combination of 17% EDTA as the chelating agent and PUI as the activation modality obtained better results than XP and in the coronal part of the canal, PUI showed better results than XP regardless of the chelating solution used. These results may reinforce the hypothesis that the possible contact of the XP with the root canal walls may reduce the elimination of the smear layer. 

Furthermore, in a recent study [ 46 ], XP was shown to be superior to positive pressure and sonic activation but similar to PUI in the elimination of calcium hydroxide from artificial internal resorptions. This fact may be due to its transformation to the A-phase, which makes the file to spread out adapting to the irregularities of the root canal [ 25 ]. In agreement with the manufacturer claims, Leoni *et al*. [ 47 ] reported that XP removed almost 90% of accrued hard tissue debris in mandibular molars due to the alloy properties, the reduced core size and no taper. Furthermore, Elnaghy *et al*. [ 33 ] demonstrated the efficacy of XP in removing smear layer and debris from curved root canals.

Similar to this study, Zand *et al*. [ 34 ] analyzed the efficacy of XP in smear layer removal with different solutions. They concluded, in accordance with our work, that XP promotes smear layer removal in combination with NaOCl and EDTA. However, there are several methodological differences, as they did not use a closed system or a flow rate standardization device and did not compare XP with other activation systems. On the other hand, De-Deus *et al*. [ 31 ] showed no differences between PUI and XP in the removal of debris, but without the consideration of the usage of weak chelating agents during instrumentation or the activation of strong chelating agents such as EDTA in the final irrigation. The activation time for this study was 30 seconds for EDTA and NaOCl based on previous reports [ 36 , 48 ]. Similarly, the NaOCl and HEDP mixture was activated for 60 seconds but refreshed after 30 seconds. 

The finding found in this study showed that, regardless of the irrigation protocol used, the coronal part of the canal was always cleaner than the apical and middle thirds. Some studies agree with this discovery and could be attributed to the larger diameter of the canal in the coronal third, which exposes dentine to a higher volume of irrigants and facilitating making smear layer removal by increasing the effectiveness of the activation systems [ 49 ]. 

## Conclusion

In summary, using a standardized flow rate of 3mL/min in combination with 17% EDTA as the chelating agent and PUI as the activation modality was significantly more effective in the removal of the smear layer from the root canal walls, but none of the irrigation methods assessed was able to completely remove the smear layer.

## Conflict of Interest

The authors have declared that no competing interests exist.
